# Use of portfolios in teaching communication skills and professionalism for Portuguese-speaking medical students

**DOI:** 10.5116/ijme.5e2a.fa68

**Published:** 2020-02-14

**Authors:** Renato Franco, Camila Ament Giuliani Franco, Marco Antonio de Carvalho Filho, Milton Severo, Maria Amelia Ferreira

**Affiliations:** 1 Medicine School, Pontifical Catholic University of Paraná, Curitiba, Brazil; 2 Center for Education Development and Research in Health Professions - Research Group LEARN - Lifelong Learning, Education & Assessment Research, University of Groningen, Groningen, The Netherlands; 3 Department of Medical Education and Simulation, Faculty of Medicine, University of Porto, Portugal; 4 Department of Public Health and Forensic Sciences, and Medical Education, Faculty of Medicine of the University of Porto, Portugal

**Keywords:** Professionalism, communication, portfolio, medical education, reflection

## Abstract

**Objectives:**

This study aimed to analyse the effect of a portfolio with three activities fostering students’ reflection, self-efficacy and teaching of communication skills and professionalism.

**Methods:**

A cross-sectional study was applied with a sample of third- and fourth-year medical students in one Portuguese and three Brazilian universities. A three-activity portfolio (course evaluation and learning, self-efficacy activity and free reflective writing) was used during a two-month course on communication skills and professionalism. The 69 students enrolled in the course were invited to complete the three-activity portfolio via Likert-type questionnaires, open-ended questions and narrative. Content and lexical analysis and the Reflection Evaluation for Learners’ Enhanced Competencies Tool (REFLECT) were used for assessing the qualitative data. The questionnaires were evaluated using principal components analysis and Cronbach’s α. Pearson’s correlation was applied to portfolio activities.

**Results:**

Of the 69 participants, 85.5% completed at least one activity. Reflecting on what they learned in the communication module, the students did not mention professionalism themes. In the self-efficacy activity on communication, 25% of the fragments were related to professionalism themes. There was a negative correlation between students’ self-efficacy and the REFLECT rubric score (r_(19)_=−0.744; p< 0.0001).

**Conclusions:**

Teachers must consider the activity’s influence on the reflections when assessing the portfolio. This model of a three-activity portfolio provided diverse ways of encouraging and assessing reflections, supporting teaching improvement and adaptation, evaluating students’ self-efficacy and showing that students’ higher reflective capacity may promote feelings of low effectiveness.

## Introduction

Communication skills and professionalism must be considered competences developed longitudinally from the beginning of medical training.^1^^,^^2^ Curricular approaches utilising reflective teaching with portfolios are increasingly employed in medical education, and such methods have been applied for communication and professionalism teaching.^3^ However, an inadequate portfolio design may not generate reflection and may promote rejection regarding its use.^4^^,^^5^ The structure of a portfolio can facilitate the promotion of students’ self-awareness, integrating theory with practice and reflection.^3^^,^^6^ Thus, the decision on the use of portfolios must be made along with the careful design of their structure and tasks to facilitate and support students’ reflections and amplify portfolio’s use.^6^ Evaluation of the use of portfolios and activities is necessary to determine their applicability and usefulness in teaching and learning.

The term portfolio is broad, and its definition needs to consider the purpose of the portfolio’s application.^7^ Applied as a teaching tool, a portfolio can be considered as a collection of learning outcomes (i.e. texts, learning journals, diaries, narratives and videos) presented for a teaching and learning purpose and associated with students’ intellectual engagement (mostly reflection).^3^ In portfolios for learning purposes, reflection has been highlighted as an essential element to characterise the material as a portfolio.^8^

Reflection can be understood as a metacognitive process (‘thinking about thinking’) focussed on a deep understanding of the situation and the self who is reflecting.^9^ Writing is the most often used method for stimulating student reflection; Wald and colleagues developed one of the main applied methods for assessing medical students’ reflective writing.^10^^,^^11^ The Reflection Evaluation for Learners’ Enhanced Competencies Tool (REFLECT rubric) was designed for reflective writing assessments, and it comprises five obligatory elements where the subject must describe a given situation (‘writing spectrum’), include him or herself as one of the elements inserted in the resolution of a problem (‘presence’), identify a dilemma or dilemmas (‘disorienting dilemma’), be aware of and consider the emotions and affect involved (‘attending to emotions’) and find meaning for him or herself and others about the experience or situation experienced (‘meaning making’).^11^^,^^12^ Communication skills and professionalism are the main themes reflected on by medical students,^13–15^ and reflection seems to be a good teaching strategy for the development of these medical competencies.^16^

Communication skills and professionalism are core competencies in medical education, related to the dialogue between the doctor, healthcare team, patients and society and helping achieve clinical goals and trust in healthcare.^17–19 ^In decisions regarding communication and professionalism, the physician needs to adapt his or her practice to different contexts and patients, and through reflection, consider different perspectives and make more appropriate decisions.^11^^,^^12^ Considering different perspectives includes the recognition of medical students’ personal perspectives and capacities. Reflecting on their capacities (self-efficacy) is important for the development and improvement of skills.^20^ Thus, developing reflective strategies in the portfolio would foster students’ ability to know and analyse their capacities, which could improve the learning of communication and professionalism.

Self-efficacy can be defined as the subject’s beliefs on his or her capacities to achieve results or behave effectively, and it is related to the achievement of better outcomes in communication skills.^21^ The association between self-efficacy and reflection is not well studied, but both these elements can influence learning.^22^^,^^23^ Reflection stimulates students to deepen their evaluation about a situation or problem, while self-efficacy beliefs are related to one’s capacity to perform a task or solve a problem. Understanding and critically viewing a situation (reflection) and one’s capacity to perform (self-efficacy) can help with both competent communication skills and professionalism. Thus, the presence of reflection and self-efficacy could enhance the quality of a portfolio’s content and expand the applicability of the portfolio as a teaching strategy.

The evaluation of teaching activity (course) is essential for better learning outcomes.^24^ The students’ evaluation of the learning is useful for remediating, adapting and improving the teaching.^25^ Moreover, the students’ perceptions on what they already knew and what, how and why they learned can provide a reflection on the teaching and learning process, engaging the students and making this process meaningful.^26^^,^^27^

The use of a reflective portfolio can be beneficial for the teaching of medical professionalism and communication skills. However, this raises the following question: What type of format and structure should be used in portfolios to support the students’ learning process? We investigated whether a portfolio incorporating three different activities can succeed in stimulating reflection about the students’ communication skills and professionalism; we also aimed to provide insight into the design of tasks that may support the use of portfolios, including reflection, self-efficacy and teaching and learning evaluation.

## Methods

### Design and study participants

A convenience sample of medical students was recruited from four universities (three in Brazil and one in Portugal). All the participants were volunteer medical students in their third or fourth year of medical school. For the sample recruitment, a class representative of the students in the third or fourth year sent an email to their colleagues inviting them to participate in the course. No financial incentives were given for their participation.

In total, 69 students from the following sites participated: 20 at the first Brazilian university (two groups of 10), 12 at the second Brazilian university (one group), 30 at the third Brazilian university (two groups of 15) and 7 at the Portuguese university (one group)’. Furthermore, 69.6% of the participants were in their fourth year and 30.4% in third year, 79.7% were female, and the mean age was 23.5 years (standard deviation [SD]: 2.495 years).

This research was approved by the Ethics Centre of the São João Hospital Centre of the Faculty of Medicine of the University of Porto and the Research and Ethics Commission of the Pontifical Catholic University of Paraná. The research objectives, procedures and risks/benefits were explained to all the participants, and they signed a consent form prior to the participation in the study.

A portfolio was applied during the course on clinical communication and professionalism as a task to be performed between face-to-face meetings. The course took place at one Portuguese and three Brazilian universities, lasted two months and consisted of five meetings for the communication module (25 hours) and four meetings for the professionalism module (16 hours). There were two face-to-face classes every two weeks, one regarding communication and the other regarding professionalism. The same two instructors conducted the course across all four universities. After the first class, the students received a weblink for the portfolio with an instruction to complete the activities in two months.

The course was not part of the formal curricula of the medical schools participating in the study. Thus, the students who chose to participate in the course also had to keep up with their curricular activity.

### Data collection methods

We developed and applied a portfolio composed of three tasks, namely, course evaluation and learning (CEL), self-efficacy activity (SEA) and free reflective writing (FRW). The data of this study were collected from the activities of the student’s online portfolio, the platform of the portfolio was designed using the Qualtrics software. The portfolio was anonymous, and the activities were not obligatory for the achievement of the course. The portfolio was intended to be carried out as a distance activity without using the time of the face-to-face meetings. Moreover, the portfolios were read, coded and assessed by two investigators (CAGSF and RSF).

### Measurements and assessment tools

#### Course Evaluation and Learning (CEL)

The CEL activity was assessed using a six-item questionnaire, where the items were rated on a Likert-type scale (1= strongly disagree, 5 = strongly agree). Higher scores indicated greater student appreciation of the course (Course Evaluation Questionnaire [CEQ]; Table 1). Two open-ended questions for reflection were also included (‘What did you learn in the modules [professionalism and/or communication]?’ and ‘What did you want to learn but were not taught, and do you have any other suggestions for the professionalism or communication modules?’). The Cronbach’s α coefficient of the CEQ was 0.805. The principal components analysis showed that the first component explained 52.39% of the total variance, with factor loadings ranging from 0.64 to 0.85. Thus, the final score of the CEQ was calculated as a mean of the six items.

#### Self-efficacy activity (SEA)

In the SEA, the participants were asked to assess their self-efficacy in a clinical task (Self-Efficacy Questionnaire [SEQ]), such as interviewing inpatients or in ambulatory and primary care settings, using an eight-item questionnaire, where the items were rated on a Likert-type scale (1=strongly disagree, 5=strongly agree). Higher scores indicated greater student self-efficacy in communication skills and professionalism. The questionnaire was based on the Clinical Communication and Professionalism Questionnaire of Capability (CCPQC).^21^ The SEQ was followed by an open-ended question: ‘Why did the clinical/academic task stimulate the development of communication or professionalism?’ The objective of the SEA was to assess the students’ self-efficacy and stimulate reflection on how the clinical or academic setting could foster the development of communication skills and professionalism. The Cronbach’s α coefficient of the SEQ was 0.764. The principal components analysis showed that the first component explained 52.8% of the total variance, with factor loadings ranging from 0.37 to 0.87 (Table 1). The SEQ score was calculated as a mean of the eight items.

**Table 1:** Validity of the Questionnaires in the Portfolio Activities

Activity	Questionnaire	No. Items	Factor Load		Cronbach’s alpha	One component variance*	Second component variance*
			Lower	Higher			
Course Evaluation and Learning (CEL)	Course Evaluation Questionnaire	6	0.641	0.858	0.805	52.39%	16.13%
Self-Efficacy Activity (SEA)	Self- Evaluation Questionnaire	8	0.592	0.879	0.764	52.8%	19.6%
Free Reflective Writing (FRW)	REFLECT rubric	5	0.639	0.904	0.850	64.1%	18.8%

*The second variance component expresses how much the addition of one more component contributes to explaining the total variance of the questionnaire. The factor loading was calculated using principal components analysis.

#### Free Reflective Writing (FRW)

For the FRW activity, the participants were asked to write a reflective narrative, and they were given some advice about the writing method (e.g. ‘describe the context’, ‘highlight main points for discussion’, ‘insert your opinion and what you felt in or about the situation’, ‘insert other authors’ opinions or theoretical references (if appropriate)’ and ‘summarise your thoughts and ideas’). At the same time, the students were free to choose the theme or situation they wanted to write about. The FRW was assessed using the Reflection Evaluation for Learners’ Enhanced Competencies Tool (REFLECT).^11^^,^^12^ Among the tools for the assessment of reflections, the REFLECT rubric is one of the most frequently used according to the Best Evidence in Medical Education Guide 51.^10^ The REFLECT rubric is an assessment guide designed to assess written reflection, and it consists of two axes.^11^^,^^12^

**Table 2:** Proportion of Participation in the Free Reflective Writing (FRW) Activity According to the University

Portfolio Activity	Participation	University					p-value (chi-square**)
		1	2	3	4	Total	
		n (%)	n (%)	n (%)	n (%)	n (%)	
Subjects Achieving FRW	yes	10(50)	6(50)	6(20)^*^	5(71.4)^*^	27 (39)	0.026^3^
	no	10(50)	6(50)	24(80)	2 (28.6)	50 (61)	

University 1, 2 and 3 are the Brazilians universities and University 4 is the Portuguese University.^*^The difference was between the frequency of achievement between University 4 and University 3; ^**^The p-value was considered statistically significant when lower than 0.05.

**Table 3.** Number of Fragments and Frequency of Thematic Subcategories in Portfolio Activities

Questions in the portfolio activities		Communication Subcategories					Common	Professionalism Subcategories			Total
		NV^*^	PP^*^	SC^*^	CDS^*^	DPR^*^	R^*^	ER^*^	EA^*^	V^*^	
		n (%)	n (%)	n (%)	n (%)	n (%)	n (%)	n (%)	n (%)	n (%)	n (%)
Course Evaluation and Learning											
	What did you learn in the communication module?	13 (19)	8 (11)	15 (21)	9 (13)	9 (13)	16 (23)	0	0	0	70 (100)
	What did you learn in the professionalism module?	0	0	0	0	0	37 (58)	12 (19)	9 (14)	6 (9)	64 (100)
	IRAMUTEC analysis	skills, listening, and summarising						reflection, empathy, ethics, respect and attitude			
Self-Efficacy Activity											
	Why did the clinical activity stimulate the development of communication?	4 (10)	10 (25)	6 (15)	2 (5)	3 (7.5)	5 (12.5)	0	9 (22.5)	1 (2.5)	40 (100)
	Why did the clinical activity stimulate the development of professionalism?	1 (2)	7 (15.5)	0	0	1 (2)	8 (18)	10 (22)	12 (27)	6 (13.5)	45 (100)
	IRAMUTEC analysis	understanding, orientation, context, and clinic						reflection, thinking, respect and ethics			
	Free Reflective Writing	11 (8)	20 (13.5)	16 (10)	0	21 (14)	^**^	26 (17.5)	31 (21)	24 (16)	149 (100)
	IRAMUTEC analysis	to put in’, ‘stay’, and ‘patient’									

^*^Non-verbal communication (NV), the patient perspective (PP), the steps of consultation (SC), communication in difficult situations (CDS), doctor-patient relationship (DPR), reflection (R), ethics and responsibility (ER), empathy and altruism (EA) and values (V). The numbers are related to how many times the subcategory occurred in each question. The number of times is given first and the frequency, relative to all fragments of the same question, is given in parentheses.^**^All fragments involved reflection in FRW.

Axis I has five criteria, namely, the writing spectrum, presence, description of conflict or disorienting dilemma, attending to emotions and analysis and meaning-making. Each of these items can be scored from 1 to 4 (a higher score means deeper reflections). Axis II has one criterion that assesses the presence of transformative learning for critical reflection and is scored from 0 to 2 (no learning, 0; confirmatory learning, 1; transformative reflection and learning, 2).^11^^,^^12^ As the original study, the score (REFLECT rubric score [RS]) is composed by the Axis I mean of the items; the Axis II was a confirmatory item and did not form part of the score. A positive correlation was expected between Axis I and Axis II. The Cronbach’s α coefficient of the RS was 0.850. One component explained 64.1% of the total variance, with loadings of 0.63–0.90 (Table 1). The FRWs were assessed independently by two assessors, and the intra-class correlation of the RS was 0.918 (p < 0.0001).

#### Portfolio Appreciation (PA)

The student appreciation of the portfolio use was measured by the Portfolio Appreciation (PA) questionnaire on a five-item Likert-scale questionnaire (1=strongly disagree, 5= strongly agree). Higher scores indicated greater student appreciation. The questionnaire was composed of student perspectives on the experience of using the portfolio and its capacity to improve learning, promote reflection of the practice, and demonstrate students’ strengths and weaknesses. The students responded to this questionnaire at the end of the course; the Cronbach’s α was 0.910, and the factor loadings were between 0.828 and 0.894.

### Statistical analysis

The portfolio was composed of three activities. The CEL activity and SEA comprised Likert-type scale questionnaires and open-ended questions, while the FRW activity comprised a narrative. Thus, we provided analysis for quantitative (doctor-patient) and qualitative data (open-ended questions and narrative).

### Qualitative analysis

**Figure 1:**
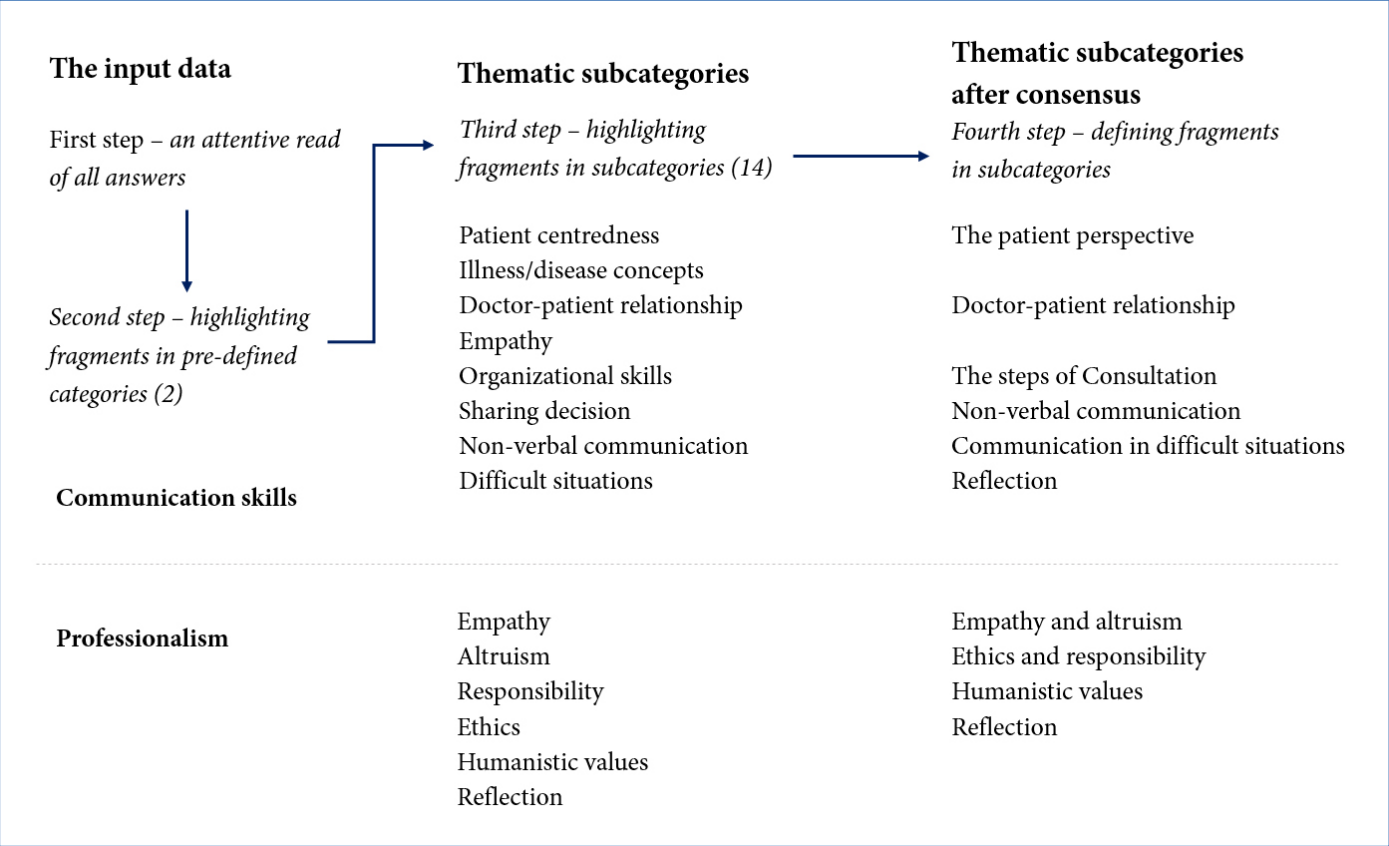
Content analysis and categories

Content and lexical analysis were applied for all the open-ended questions and narratives. The content analysis,^28^ supported by the NVivo software, was used to find thematic categories and subcategories. Two readers conducted the analysis independently and established a consensus during two meetings. All open-ended questions, and the narrative, were analysed for content using two pre-defined thematic categories (professionalism and communication), and the subcategories were identified according to the content of the answers. After the definition of the subcategories, all the answers were analysed again to determine the presence or absence of the subcategories (Figure 1).

The lexical analysis was performed using the Iramutec, an R interface software for the multidimensional analysis of texts and questionnaires (e.g. word frequency). The software uses the Reinert method,^29^ a factorial analysis where all the words are put together in a single cluster and divided according to chi-square criteria for the separation of words into classes.^29^^-^^31^

**Table 4:** Univariate Linear Regression Analysis for the Effect of Demographic Variables in Portfolio Activities

Variable	Scores in the Portfolio Activities											
	CEQ Score (n=50)				SEQ Score (n=36)				RS (n=27)			
	B	SE	β	p	B	SE	β	p	B	SE	β	p
Gender												
Female	0.019	0.112	0.060	0.868	0.346	0.181	0.822	0.056	0.070	0.281	0.109	0.802
Male	ref				ref				ref			
Age	−0.007	0.018	−0.051	0.712	0.017	0.042	0.101	0.681	0.017	0.055	0.057	0.753
University												
1	−0.184	0.169	−0.589	0.275	−0.184	0.258	−0.437	0.477	0.864	0.367	1.342	0.018
2	−0.239	0.204	−0.766	0.240	−0.022	0.306	−0.053	0.942	−0.172	0.456	−.0164	0.706
3	−0.024	.0155	−0.076	0.879	−0.044	0.259	−0.103	0.866	0.368	0.393	0.635	0.349
4	ref				ref				ref			
Academic Year												
3	0.027	0.115	0.085	0.817	−0.218	0.209	−0.517	0.298	0.501	0.320	0.688	0.117
4	ref				ref				ref			

CEQ: Course Evaluation Questionnaire; SEQ: Self-Efficacy Questionnaire; RS: REFLECT rubric score; B=regression coefficient; SE=standard error of regression coefficient; β=standardized regression coefficient. Universities 1, 2 and 3 – Brazilian Universities, University 4 – Portuguese University. The p-value was considered statistically significant when lower than 0.05.

Principal components analysis was applied to all the Likert-type questionnaires to assess the dimensionality and associated items to each component. Dimensionality was assessed using a scree plot, and the number of components that needed to be retained in each Likert-type questionnaire was assessed according to the ‘elbow rule’. An element or item was considered to contribute to a principal component when it had a correlation value higher than 0.30. The internal consistency was evaluated using Cronbach’s α.

**Table 5:** Pearson’s Correlation Coefficients Between Portfolio Activity Scores

Scores	CEQ		SEQ	
	r	p-value^*^	r	p-value^*^
RS	−.058	0.798	−.744	<0.0001
SEQ	−.030	0.893		

r: correlation coefficient; ^*^The p-value was considered statistically significant when lower than 0.05. CEQ: Course Evaluation Questionnaire; SEQ: Self-Efficacy Questionnaire and RS: REFLECT rubric score.

The chi-squared test was used for comparing the proportions between different groups. Analyses of variance (ANOVA) were used to compare the means between three or more independent groups.

The Pearson correlation coefficient and linear regression analysis were employed to find the magnitude of the linear associations. The data were analysed using the Statistical Package for the Social Sciences (SPSS), and the significance level was fixed to 0.05.

## Results

### The achievement of activities

From a total of 69 participants, 59 (85.5%) completed at least one of the portfolio activities and score representing student appreciation (n =59) on the use of the portfolio activities was 3.83 (SD: 0.710). Each student completed 1.6 activities (SD: 1.05); 36.2% completed one activity, 20.3% carried out two activities, 29% finished three activities and 14.5% did not complete any of the activities (CEL, SEA and FRW). The CEL had the highest frequency of achievement (n= 50; 72.5%), followed by the SEA (n= 36; 52.1%) and FRW (n= 27; 39.1%). The Pearson’s chi-square for CEL (χ^2^_(3, N= 69)_(3, N= 69) =6.212, p = 0.102) and SEA (χ^2^_(3, N=69)_ =5.365, p=0.147) did not demonstrate a statistically significant difference between the universities. The achievement of the FRW presented differences between the universities (χ^2^_(3,N=69)_=9.263, p = 0.026); Table 2).

### CEL

The CEQ score was 4.59 (SD: 0.412). There were 134 response fragments for the questions ‘What did you learn in the module?’ (64 on professionalism and 70 fragments on communication).

In the content analysis, the students pointed out that ethics and responsibility (ER), empathy and altruism (EA) and humanist values (V) were the themes they had learned about in the module on professionalism. In the module on communication, the students highlighted that they learned about non-verbal communication (NV), the patient perspective (PP), the steps of consultation (SC), communication in difficult situations (CDS) and the doctor-patient relationship

(DPR). The students did not attribute themes regarding professionalism to the communication skills module or vice versa. Fostering reflection (R) was pointed out for communication and professionalism as one of the strengths of the course. The frequencies of fragments in each subcategory are displayed in Table 3.

The lexical analysis clustered the words ‘reflection’, ‘professionalism’, ‘empathy’, ‘ethics’ and ‘respect’ into one class related to answers on professionalism (the chi-squared result was 4.1 to 18.8; p<0.05). The terms ‘patient’, ‘skills’, ‘talk’, ‘open-ended (questions)’, ‘well’, ‘consultation’, ‘asking’, ‘anamnesis’ and ‘communicate’ (chi-squared result in the range of 4.12–12.91; p<0.05) were clustered into the class related to communication skills (Table 3). The lexical and content analysis showed that, when students reflect on what they have learned in the modules, they delimit the concepts related to communication and professionalism without pointing out the interfaces between then.

Responses to ‘What did you want to learn but were not taught, and do you have any other suggestions for the professionalism or communication modules?’ included suggestions on learning and themes, but some responses had no suggestions. There were 64 response fragments (35 for the communication module and 29 for the professionalism module); 26 fragments were on complements regarding the modules or did not have suggestions, 21 suggested themes (e.g. including themes on confidentiality and students’ duties in professionalism, dealing with own and patient emotions in the consultation and adapting the consultation according to patients’ personality in communication); and 17 were regarding learning methods (e.g. more practical activities in professionalism and communication, and more traditional lectures in the professionalism module).

### SEA

In the SEA, the participants could assess their self-efficacy in clinical or academic activities. For SEQ, the mean score was 4.29 (SD: 0.421).

There were 45 responses answering the question, ‘Why did the clinical activity stimulate the development of communication and/or professionalism?’ (Table 3). The students reflected that the clinical activity fosters the development of communication skills; the responses could include discussion of NV, PP, SC, CDS, DPR, EA and V. The ER was not mentioned in terms of the stimuli of communication skills. For professionalism, the students pointed out NV, PP, DPR, ER, EA and V. The medical students did not include SC or CDS for the learning of professionalism (Table 3).

In the lexical analysis, the answers were clustered into two classes (one closest to professionalism and another to communication). The class related to professionalism contained the words ‘reflection’, ‘thinking’, ‘respect’, and ‘ethics’, and the chi-squared result was 14.92 to 21.0 (p<0.001). For communication, the software clustered the words ‘understanding’, ‘orientation’, ‘context’ and ‘clinic’, and the chi-squared results ranged from 4 to 31.1 (p< 0.05). The clustering by the lexical analysis provided by Iramutec for open-ended questions in SEA a CEL was similar to the subcategories of the content analysis, which reinforces the validity of the categorisation system (Table 3). When the students reflected on clinical activities and self-efficacy, they highlighted that professionalism elements could foster the development of communication skills and vice versa.

### FRW

The REFLECT rubric results of Axis I were analysed as one score called the REFLECT rubric Score (RS), which had a mean of 2.58 (SD: 0.675). The mean RSs according to Axis II was 3.24 (SD: 0.325) for transformative learning, 2.66 (SD: 0.406) for confirmatory learning and 1.50 (SD: 0.208) for neither, with significant statistical differences between the means (ANOVA - F_(2,27) _= 33.949, p<0.0001). Once Axis II works as a global rate on the REFLECT rubric, the improvement of Axis I according to the levels of Axis II reinforces the validity of the instrument.

All the narratives written involved practical situations where the students observed or participated in the clinical interview. After identifying subcategories related to professionalism and communication, all the fragments associated with these elements were grouped into one document and analysed using Iramutec. This revealed three terms with the closest relative frequencies in reflections on both communication and professionalism, that is, ‘put in’, ‘stay’ and ‘patient’. ‘Put in’ referred to putting oneself in the patient’s place (i.e. imagining the patient’s perspective). In the analysis of the FRW, the software did not cluster the fragments in groups related to communication and professionalism. The FRW was a free reflection, and themes related to professionalism and communication were involved (Table 3). It was not possible to determine whether one narrative was about communication or professionalism issues, as both elements were strongly present in all the students’ reflections.

### Factors associated with activity scores

The participants’ gender, age, academic year, and the university had no effect on the SEQ and CEQ, although in the RS, the students’ university influenced the score (Table 4). A negative correlation was observed between the SEA (self-efficacy) and the RS (r_(__19)_ = −0.744, p < 0.0001; Table 5).

## Discussion

The portfolio activities promoted a platform for reflection, especially on the themes covered by the course. All the course content was covered by the students’ writings, although the frequency of each theme was different among the activities. Each portfolio activity provided stimuli for the students to reflect on the diverse elements of communication and professionalism. This model of three activities provided three main functions—obtaining information on course evaluations, monitoring students’ learning and integrating the course on communication and professionalism with other curricular activities. The course evaluation in the electronic portfolio provides important information to professors to adapt the course according to the participants’ needs during the sessions. There was a peculiar inverse association between the SEA and scores that assessed the RS.

### ‘Just-in-time’ course evaluation

The CEL activity provided information for teachers to enable them to adapt the course according to the learning objectives. The purpose of this activity was improving the communication between the students and teachers. A systematic evaluation is an important tool to improve the quality of a course^33^ and support the development of teaching.^34^ The activities were sent electronically to the students as soon as each class ended, and this system provided feedback for professors that reinforced the importance of knowing students’ opinions ‘on time’ to promote adjustments in the next encounters.^35^^,^^36^

The CEL applied the principles of ‘just-in-time teaching’, where the teaching must be adapted to the student’s needs and learning.^37^ For example, in communication classes, students suggested that the teacher participates in the simulation so that the students could analyse and discuss the teacher’s performance. Teachers did so, and the students pointed to this as an enlightening task. We call the students’ evaluation during the modules followed by adaptations in the modules ‘just-in-time course evaluation’, and to assess this, we focussed on the students’ evaluation of the course—what they learned and what they wanted to learn in the next classes.

The open-ended questions provided information on what themes students learned and suggestions to improve the teaching; the Likert-type questionnaire was useful for determining the quality of the modules. In the evaluation of the courses, diverse methods of assessment must be applied, and Likert-type evaluations alone are not enough.^24^ Traditionally, evaluations are usually conducted at the ends of courses;^24^ however, when they are carried out during the course, this promotes the opportunity to adapt the teaching before the course ends.^26^ Thus, this activity was important for showing how to adapt the content and methods of the course, as well as illustrating the course’s weaknesses and student needs.

### Diverse stimuli to reflect

The design of the portfolio can determine the objectives and purposes of its use. For Saltman and colleagues, the reflective portfolio must include reflective and reasoned elements. Reasoned tasks are related to the demonstration of students’ understanding, finding definitions and particularities of the concepts and identification of their learning needs.^38^ The results of the CEL can then be understood as a ‘reasoned’ task. In the study, the students referred to communication and professionalism using terms and definitions that allowed them to discriminate clearly between discussing communication and professionalism.

In the CEL, it was possible to characterise the learned concepts on communication and professionalism, but in the reflection on practice (noticed in SEA and FRW), the development of these competencies merged. Asking students to reflect on self-efficacy also stimulated the discussion and understanding of communication and professionalism. However, themes on communication were pointed out to influence the development of their efficacy on professionalism and vice versa. In the FRW, students provided a deep refection based on real situations, and elements of professionalism and communication were described together to evaluate dilemmas. In the free reflection, it was impossible to distinguish the reflections on communication and professionalism once all the students analysed the situation, as they pointed out both elements. Thus, the design of the portfolio activity may influence what topic students reflect on and how.^6^ Reflecting on practical activities (as happened in FRW and SEA) fostered students’ inclusion of wide themes and diverse concepts, but the CEL showed the students’ understanding and differentiation of the concepts.

### Reflection, self-efficacy and curriculum

The high scores on self-efficacy in the SEA showed that the students must be receptive to change behaviours according to the teacher’s orientation^22^^,^^23^ and practice what was taught.^39^ The results of the REFLECT rubric were similar to the results in other studies (2.60 to 2.71),^40^^,^^41^ although the inverse correlation of the RS with the self-efficacy calls attention to the possibility that higher academic achievement^42^ and improvement of the cognitive process^43^ may make students feel less prepared in demonstrating complex behaviours such as professionalism and communication. The self-efficacy in practising a behaviour or skill in professionalism and communication does not necessarily increase the reflection or self-awareness about the situation.^44^

This reinforces the importance of not only reflection-in-action during a situation but also the promotion of opportunities to improve students’ ‘reflection on action’ after an event, which can follow a patient-care experience, class or other learning activity.^45^ It is essential to find the balance between the reflection, including the deep understanding of a situation and the complexity of solutions with the evaluation of students’ self-efficacy, revealing the challenges on providing safety for patients and supporting student confidence.

The students’ evaluation of their self-efficacy and the writing narratives stimulated reflection on clinical encounters in formal and hidden curriculum promotion and integration of the learning content on the communication and professionalism course into the curricula. One of the challenges for structuring the curriculum is to provide integration of the fragmented delivery of the knowledge (mainly in classes) to a synthesised and comprehensive application of the knowledge to the development of students’ competence.^46^ The SEA and FRW can assist this process of integration stimulating students to reflect on their capabilities and profoundly analyse clinical encounters.

## Conclusions

The assessment of the portfolio showed three complementary functions, namely, the support to teaching and learning improvement (course evaluation); stimulus for reflection, including the presentation of students’ learned concepts, knowledge and deep reflections; and the integration of the reflection with practice (self-efficacy). In the assessment of the content of students’ reflection, teachers must consider how the included activity influences students’ highlighting of different themes. In this study, the students seemed to exhibit better acceptance of more structured activities in the portfolio (in the CEL and SEA), although the contents of reflections were higher and more profound in the free narrative.

Reflective portfolios in medical education must go beyond assessing students’ reflective ability and identifying the themes on which they reflect. The inclusion of reflective activities that encourage students to reflect on their learning and skills broadens the application of portfolios, expands their use and outcomes and should be encouraged in medical education. In addition, we suggest that the portfolio should be organised in such a way that it assists the teacher in improving the teaching and learning process.

This portfolio—based on a course evaluation, self-efficacy and free narrative reflection—may be suitable for the development of reflective teaching of communication skills and professionalism. Future studies must evaluate the use of this portfolio model in other contexts and the application to other domains of competencies, such as clinical skills and medical knowledge.

### Limitations

The main limitation of this study was convenience sampling. Students were invited to participate in the courses. Thus, those who opted to participate were probably highly motivated students. The main influences of the sample bias likely involved the completion of the activities and evaluation of the course. The influence of this bias on the results concerning the portfolio could be minimised by students being highly motivated for the themes of professionalism and communication but not the method of using the portfolio. Nevertheless, the motivation on the theme certainly influenced the quality of the reflection and the themes discussed.

A previous study showed that 33% of students completed a portfolio that was non-obligatory.^47^ The completion of all activities in our study was around 50%. This is not as low as in other studies, but we expected a higher rate of portfolio completion. The subjects did not choose to participate in the course because of the portfolio but because of the course content, and almost all of them had never done portfolios before. The explanation on the completion of the portfolio was on the first page of the tasks. The students did not complain about the portfolio instructions, but we felt that clearer instructions could have been provided.

### Acknowledgement

We thank all medical students who participated in this research. Financial support for the authors was provided by scholarships from the Conselho Nacional de Desenvolvimento Científico e Tecnológico (Brazilian National Council of Technological and Scientific Development, 229753/2013-2) and the Coordenação de Aperfeiçoamento de Pessoal de Nível Superior (Coordination for the Improvement of Higher Education Personnel, Brazil, 13271/13-0).

### Conflict of Interest

The authors declare that they have no conflicts of interest.
